# The Jubilee of Anæsthetics

**Published:** 1896-10-17

**Authors:** 


### The Hospital

A Journal of

The Medical Sciences and Hospital Administration, with

AN EXTRA NURSING SUPPLEMENT.

Vol. XXI.—No. 525.] [Oct. 17, 1896.

### The Jubilee of Anæsthetics.

In the year 1846—exactly half a century ago, and for the first time in history—Dr. Morton, of Boston, employed an anæsthetic to prevent pain during a dental operation, and on October 16th of that year it was publicly used in the surgical theatre of the Massachusetts General Hospital. The present year, in fact the very day on which these words will reach our readers, is, therefore, the jubilee of the introduction of anæsthesia into medical and surgical practice. The modern world, the world of science, has many triumphs and practical gains to record. Where does the discovery and application of anæsthesia in practice stand among the gains and triumphs of knowledge over ignorance? It stands, if we mistake not, among the most practically important of all scientific discoveries—in the very front rank.

The unthinking may consider that anæsthesia in medical practice is a relatively insignificant thing because medical practice itself deals with but a small minority of the people, that is, with the sick only. But medical science and practice do not, as now understood, concern themselves with the sick only, but with the whole race of man. Medicine, in fact, as we have so often contended here, is like philosophy, it is a "science of man." Every important medical discovery and advance in practical capacity is of universal and world-wide significance. There has hardly ever been known a discovery and advance of greater importance in medicine than the discovery of surgical anæsthesia. If this discovery be so important to medicine, and if scientific medicine be so important to the whole human race, then, at least in the extent of its actual and potential beneficent application, it deserves to rank among the first and noblest of scientific discoveries.

Within their own sphere physicians and surgeons are the least imaginative and the most practical of mankind. The finest theory in the world ceases to attract them if it is found that no solid content of practicality is found within it. Let the reader for a moment recall how many circumstances of first-rate practical importance are associated with the use of anæsthetics in medicine and surgery. Naturally the most obvious and significant of all these circumstances is the anæsthetised and unfearing patient on the operation-table. All apprehension has been allayed, all terror has been banished, calm sleep has taken full possession, and instead of the struggles, and shrieks, and horror, which reigned in the operating theatre before the days of anæsthesia, the surgeon sees before him a passive organism, obedient in every respect to his will, and submissive to the sternest and most tremendous necessities of the hour. What a contrast is here between the anæsthetic and the pre-anæsthetic periods. To the patient of to-day a surgical operation has no significance at all, except such as may belong to a period of sound anæsthetic sleep. What that means compared with the surgical circumstances of the past those only know who as surgeons or patients have had personal experience of the past.

But to turn from the patient to the surgeon. However striking may be the happiness of the anæsthetised patient, as compared with the non-anæsthetised in pre-anæsthetic times, it is hardly greater than the happiness of the surgeon himself who is called upon to perform the operation. It is not merely that a cultured and humane gentleman is saved from the necessity of inflicting long and agonising pain upon a fellow-creature, it is this quite as much, that now the surgeon can set about his operation with the unflurried deliberateness which its difficulty and dangerousness demand. Now he can make it, not a mad rush to an end, which must be reached with all possible speed lest the victim die of mere shock, but a labour of art, and pains, and skill—a labour in which neither deftness nor time need be spared to bring it to desired and perfect result.

Almost more important than either of these circumstances, when looked at from the point of view of mankind as a whole, is the extraordinary enlargement of the area of surgical activity as the result of the introduction of anæsthetics. Before anæsthetics a surgical operation was generally decided upon for this sole reason, that it alone offered a chance of saving limb or life. Surgical operations were indeed, of necessity, as limited in number as possible. Now, with anæsthetics in use, an operation is decided upon, not merely because there is imminent peril to life or limb, but for any and every purpose which may be of real service to the patient. The field of surgical usefulness has thus been enlarged a hundredfold.

Another consideration of hardly inferior weight to any of the foregoing is the fact that, with anæsthetics in use, a class of men have espoused the surgical art, and continue to adopt it in ever-increasing numbers, who, under the older conditions of surgical practice, would not have been willing—would not, indeed, have been able—to practice surgery at all. The pre-anæsthetic surgeon was often spoken of as a "butcher"; and the term was in those days hardly one of disparagement. If the surgeon was a cultured man, with the skill of the competent butcher thrown in, so much the better both for himself and the patient. But the days of "swift" operations are over, never, we may hope, to return. Now, thanks entirely to anæsthetics, the surgeon is not a "butcher," but an "artist"; a skilled user of the finest tools—of tools which can be manipulated without any distracting thought, and employed with the calm deliberateness needed to secure the highest possible result to which the scientific conservation of life and structure, and function can by any possibility attain. Surgery, under these circumstances, attracts to its ranks and retains in its service men, not merely of the highest scientific capacity, but of the most brilliant imaginativeness and the most sensitive tenderness of disposition and character. The gain to the surgical art, and of course also to the whole world of the sick and suffering, is in this respect almost incalculable. How much more might be said on this wide and intensely human subject, the least imaginative of professional, and even of non-professional, readers may perceive. Reasoning on these lines, we think we are more than justified in the general contention that the discovery and utilisation in universal practice of surgical anæsthesia is one of the greatest and most beneficent of all the conquests which an all-progressive science has ever achieved.

